# Immunogenicity and Neutralization of Recombinant Vaccine Candidates Expressing F and G Glycoproteins against Nipah Virus

**DOI:** 10.3390/vaccines12090999

**Published:** 2024-08-31

**Authors:** Seo Young Moon, Rochelle A. Flores, Min Su Yim, Heeji Lim, Seungyeon Kim, Seung Yun Lee, Yoo-kyoung Lee, Jae-Ouk Kim, Hyejin Park, Seong Eun Bae, In-Ohk Ouh, Woo H. Kim

**Affiliations:** 1 Division of Vaccine Development Coordination, Center for Vaccine Research National Institute of Infectious Diseases, Korea National Institute of Health, Korea Disease Control and Prevention Agency, Cheongju-si 28159, Chungcheongbuk-do, Republic of Korea; msy1477@korea.kr (S.Y.M.); yms37@korea.kr (M.S.Y.); dalgi0519@korea.kr (H.L.); hatmddus135@korea.kr (S.K.); leeykyoung@korea.kr (Y.-k.L.); jin2147@gmail.com (H.P.); 2 College of Veterinary Medicine & Institute of Animal Medicine, Gyeongsang National University, Jinju 52828, Gyeongsangnam-do, Republic of Korea; floresrochellea@gmail.com (R.A.F.); seungyun0218@gnu.ac.kr (S.Y.L.); 3 Molecular Immunology, Science Unit, International Vaccine Institute, Seoul 08826, Republic of Korea; jokim@ivi.int (J.-O.K.); seongeun.bae@ivi.int (S.E.B.)

**Keywords:** Nipah virus, recombinant vaccine, antigenicity, pseudotype neutralization assay

## Abstract

Nipah virus (NiV), of the Paramyxoviridae family, causes highly fatal infections in humans and is associated with severe neurological and respiratory diseases. Currently, no commercial vaccine is available for human use. Here, eight structure-based mammalian-expressed recombinant proteins harboring the NiV surface proteins, fusion glycoprotein (F), and the major attachment glycoprotein (G) were produced. Specifically, prefusion NiV-F and/or NiV-G glycoproteins expressed in monomeric, multimeric (trimeric F and tetra G), or chimeric forms were evaluated for their properties as sub-unit vaccine candidates. The antigenicity of the recombinant NiV glycoproteins was evaluated in intramuscularly immunized mice, and the antibodies in serum were assessed. Predictably, all homologous immunizations exhibited immunogenicity, and neutralizing antibodies to VSV-luciferase-based pseudovirus expressing NiV-GF glycoproteins were found in all groups. Comparatively, neutralizing antibodies were highest in vaccines designed in their multimeric structures and administered as bivalent (GMYtet + GBDtet) and trivalent (Ftri + GMYtet + GBDtet). Additionally, while all adjuvants were able to elicit an immunogenic response in vaccinated groups, bivalent (GMYtet + GBDtet) and trivalent (Ftri + GMYtet + GBDtet) induced more potent neutralizing antibodies when administered with oil-in-water nano-emulsion adjuvant, AddaS03. For all experiments, the bivalent GMYtet + GBDtet was the most immunogenic vaccine candidate. Results from this study highlight the potential use of these mammalian-expressed recombinant NiV as vaccine candidates, deserving further exploration.

## 1. Introduction

A rapid and effective vaccine response strategy for outbreaks or pandemics requires both accurate antigen design and a method for rapid deployment and manufacturing. As part of pandemic preparedness efforts, we selected Nipah virus (NiV) as a prototype paramyxovirus pathogen to optimize antigen design, analyze the immune responses to vaccination, and identify mechanisms of protection. NiV is an enveloped, non-segmented negative-strand RNA virus belonging to the Paramyxoviridae family in the genus Henipavirus with Hendra virus [1]. NiV is listed as a high priority pathogen by the Centers for Disease Control and Prevention (CDC), World Health Organization (WHO), and the Coalition of Epidemic Preparedness Innovations (CEPI), and there is a need for medical counter-measures, especially vaccines [2].

The virus first emerged in late September 1998 in Malaysia when human deaths associated with severe febrile encephalitis and respiratory illness in pigs were reported. Subsequently, respiratory and encephalitis cases from abattoir workers who handled pigs from the outbreak’s regions in Malaysia were reported in Singapore in March 1999 [1]. The 1998/1999 outbreak recorded a total of 265 encephalitis cases, with 105 deaths and over a million pigs culled [1,3]. Since then, no further outbreak was reported until 2001 when NiV re-emerged in India and Bangladesh, where seasonal outbreaks continue to occur with the most recent human fatality reported in both countries in 2023 [4,5,6]. A suspected case was also reported in the Philippines in 2014, characterized by fatality in humans presenting acute encephalitis syndrome and sudden death, and concurrent neurologic disease in horses [7].

Fruit bats or flying foxes, mainly *Pteropus* spp, are identified as the natural source of NiV, and infected bats are generally asymptomatic but can spread the virus through their saliva, urine, semen, and excreta [8,9]. The genus *Pteropus* consists of about 60 species of bats that are distributed globally from the western Indian Ocean islands (Mauritius, Madagascar, and Comoro), Himalayan region (Pakistan and India), Southeast Asia (Philippines, Indonesia, and New Guinea), and the south-west Pacific Islands (Cook Islands and Australia) [10]. To date, NiV outbreaks have been reported mainly in Malaysia, Singapore, India, and Bangladesh, however, neutralizing antibodies against NiV have been detected, with some NiV isolates identified and characterized in various fruit bat species from Cambodia, Thailand, Indonesia, China, East Timor, Madagascar, Papua New Guinea, and Ghana, highlighting the possible high risk of transmission and its potential to spread [11,12,13,14,15,16,17].

The transmission of NiV to susceptible domestic livestock hosts has been associated with direct contact with infected bats or the ingestion of other contaminated fruits [9]. In humans, virus transmission has been reported to be due to direct contact with infected bats and infected intermediate host (e.g., pigs, cows, goats, horses, dogs, and cats), consumption of contaminated food products (e.g., date palm juice and meat), or person-to-person transmission through exposure with respiratory secretion or direct contact with bodily fluids [7,8,18,19,20,21,22]. The spread of NiV from the previous outbreak in Malaysia and Singapore was reported to be mainly from bats to pigs, then from pigs to humans, and an occasional human-to-human transmission. On the contrary, human-to-human spread was important in outbreaks from Bangladesh and India, along with the bats-to-human route through consumption of contaminated food products and possibly through bat-domestic animal-human spread [3,4].

Genetic analysis of NiV isolates from all the outbreaks known to cause infection in humans identified two major genetic lineages that are phylogenetically different, the NiV-MY (NiV Malaysia) strain and the NiV-BD (NiV-Bangladesh) strain. Genome length of NiV-MY and NiV-BD are 18,246 nt and 18,252 nt, respectively, with an overall nucleotide homology of 91.8% [3,23]. Similar to other paramyxoviruses, the NiV genome is unsegmented and encodes for six proteins to form the virion structure (nucleoprotein, N; phosphoprotein, P; matrix protein, M; fusion glycoprotein, F; attachment glycoprotein, G; large protein or RNA polymerase protein, L) with an additional three proteins from the P gene for accessory functions (V, C, W protein), and the genes are organized in the genome in following the order 3′N-P-M-F-G-L-5′ [24]. Infection by NiV of the host cell is facilitated by G and F surface glycoproteins, with the initial binding of G glycoprotein to host receptors (ephrin-B2 and -B3), then the fusion of the viral envelope with host membrane as mediated by F glycoprotein [25], taking place.

Analogous to other paramyxoviruses, these two surface glycoproteins are known targets of neutralizing antibodies and, thus, are the primary focus of most vaccine developments. Several vaccines using a recombinant adeno-associated virus vaccine (expressing NiV-MY-G), a VSV (vesicular stomatitis virus)-vectored vaccine (expressing NiV-BD-G), a poxvirus-vectored vaccine (expressing NiV-G or F), a measles virus-vectored vaccine (expressing NiV-G), a rabies virus-vectored vaccine (expressing NiV-G), a messenger RNA (mRNA-1215) vaccine (encoding NiV-MY-F and G), a virus-like particle vaccine (VLP-based NiV-G, F, M), a vaccinia-based vaccine (expressing NiV-G or F), and a virus replicon particle vaccine (VRP lacking fusion gene) have been shown to be effective in animal models [26,27,28]. Additionally, epitope-based NiV vaccine candidates have been designed and validated in silico to be potential candidates [29]. Recently, nucleoside-modified mRNA vaccine encoding NiV-G were also found to induce potent antigen-binding and virus-neutralizing antibodies after booster immunization in pigs [30]. However, despite the growing amount of research on NiV vaccine candidates, none is licensed for human use to date.

Structure-based design antigens have been previously demonstrated to induce high levels of immunogenic NiV sub-unit protein vaccine. Structurally, G glycoprotein is a homotetrameric type II transmembrane domain, while F glycoprotein is a trimeric type I transmembrane protein, and both have been proposed to undergo conformational changes upon receptor engagement to promote membrane fusion [31]. The fusion (F) glycoprotein utilizes a class I fusion glycoprotein known for transitioning from a metastable pre-fusion conformation (pre-F) to a stable post-fusion conformation (post-F), and to fuse viral and cellular membranes and initiate viral entry [32]. Recent reports have shown that structure-based design and engineered mutations to stabilize the pre-fusion conformation of the glycoprotein in Parainfluenza Virus (PIV) and Respiratory Syncytial Virus (RSV), and the pre-fusion of Betacoronavirus spike protein, have resulted in a more immunogenic and potent neutralizing antibody response [33,34,35].

NiV is classified as a biosafety level 4 (BSL-4) pathogen with high zoonotic potential, broad species tropism, and has caused outbreaks resulting in a case fatality rate of 39.6% (NIV-MY) for up to 40–75% (NiV-BD) depending on the strain. Recently, two genetically distinct novel paramyxoviruses—Gamak virus (GAKV) and Daeryoun virus (DARV)—were reported in the Republic of Korea. These viruses belong to henipaviruses within the family Paramyxoviridae, and GAKV showed the upregulation of multiple human innate anti-viral genes in vitro, and underscored the zoonotic potential of novel paramyxoviruses [36]. Given the severity of NiV infection and its continuous re-emergence, along with the emergence of novel paramyxoviruses, it is imperative to continuously pursue the development of safe and effective vaccine candidates as part of our pandemic preparedness efforts. In this study, a structure-based design of NiV virus was used to develop a prototype paramyxovirus pathogen to optimize antigen design. Here, vaccine-targeting NiV-F and NiV-G glycoprotein, based on a consensus sequence of publicly available NiV sequences, was designed and constructed. In total, eight mammalian-expressed recombinant proteins harboring NiV-pre-F and/or NiV-G glycoproteins in monomeric, multimeric, or chimeric forms were developed. Furthermore, the induction of primary humoral response and efficacy of the NiV-vaccine candidates was evaluated using the VSV-luciferase pseudovirus system.

## 2. Materials and Methods

### 2.1. Cell Culture

HD-293F cells and Vero cells were grown and maintained in high-glucose Dulbecco’s modified Eagle’s medium (DMEM, Gibco, Grand Island, NY, USA), supplemented with 10% Fetal Bovine Serum (FBS, Gibco, Grand Island, NY, USA), penicillin (100 IU/mL, Cytiva, South Logan, UT, USA), and streptomycin (100 µg/mL, Cytiva, South Logan, UT, USA) in a 37 °C incubator under 5% CO_2_. BHK21 cells were cultured in DMEM with 5% FBS, but no antibiotic–antimycotic agent in the same incubator condition.

### 2.2. Recombinant Vaccine Design and Phylogenetic Analysis

For the vaccine design, we used a focused consensus strategy to design NiV-F and -G glycoprotein antigen. The consensus sequences were designed through the alignment of multiple F and G glycoprotein sequences available in the PubMed database, including the two genetically categorized genotypes of NiV-Malaysia identified in Malaysia and Cambodia (NiV-M clade), and NiV-Bangladesh (NiV-B clade) identified in Bangladesh and India. A total of 78 and 71 full-length NiV surface gene sequences for F and G glycoproteins, respectively, including both strains, from Malaysia and Bangladesh, were downloaded from NCBI and collected between 1998 and 2021, but the sequences with partial open reading frame (ORF) were excluded (Table S1). In order to identify the phylogenetic groups, all sequences were converted to the FASTA format, and sequence alignment was performed using CLC Main Workbench package (ver. 7.0.3; CLC Bio, Aarhus, Denmark). A phylogenetic tree was performed by MEGA ver. 7.0 (https://megasoftware.net/, accessed on 1 April 2022) and using the HKY model [37]. A bootstrap percentage for 1000 replicates was used for statistical assessment of the generated tree. All positions with less than 95% site coverage were eliminated. Next, the reference sequences from each sub-group were selected and used to generate the consensus sequence glycoprotein.

### 2.3. Plasmid Preparation and Expression of Recombinant Proteins

A total of eight candidates harboring F or G glycoprotein based on their consensus or ancestor sequences analyzed above were selected and used to design constructs for vaccine immunogenicity experiments (Table 1). The selected sequences of pre-F or -G glycoprotein of NiV for mammalian expression were synthesized from Genscript (Nanjing, China) and cloned into pcDNA 3.4 by using restriction enzymes EcoRI and HindIII at 5′ end and 3′ end, respectively. Recombinant glycoproteins expressing NiV surface antigens were expressed using the High Density (HD) Transient Expression system (Genscript, Nanjing, China) based on suspension-adapted human embryonal kidney (HEK)-293F cells (HD-293F cells). Briefly, 30 μg of transfection-grade plasmid was transfected into 30 mL HD-293F cells at a cell density of approximately 5 × 10^6^ cells/mL, and the cells were incubated at 37 °C with 5% CO_2_ for 72 h. Then the supernatant was collected, centrifuged at 3000 rpm for 10 min, and filtered through a 0.22 μm filter (Sigma-Aldrich, St. Louis, MO, USA). The purification of 6x-His tagged recombinant proteins was performed by using High Affinity Ni-Charged Resin (Genscript, Nanjing, China). The concentration of the recombinant proteins was measured by Bradford assay (Thermo Fisher Scientific, San Diego, CA, USA) and the purity was confirmed by SDS-PAGE and size exclusion HPLC.

### 2.4. SDS-PAGE and Western Blot

All samples were mixed with an equal volume of sample buffer (0.125 M Tris–HCl (pH 6.8), 4.0% SDS, 20% glycerol, 10% 2-mercaptoethanol, and 0.004% bromophenol blue) and heated at 95 °C for 5 min. Each protein (2 μg/lane) was resolved on a 15% SDS-polyacrylamide gel and electroblotted onto nitrocellulose (Immobilon-P, Millipore, Bedford, MA, USA). The membrane was blocked with blocking buffer (5% bovine serum albumin in PBS-T), washed with PBS-T, and incubated with mouse anti-his monoclonal antibody (ThermoFisher Scientific, San Diego, CA, USA). Bound mAbs were incubated with horseradish peroxidase (HRP)—conjugated rabbit anti-mouse IgG secondary Ab (1/1000 dilution, Sigma-Aldrich, St. Louis, MO, USA), visualized using a Clarity Western ECL Substrate (Bio-Rad, Hercules, CA, USA), and detected using the ChemiDoc imaging system (Bio-Rad, Hercules, CA, USA).

### 2.5. Immunization of Mice

Six-week-old, specific pathogen-free (SPF), male BALB/c (AnNCrlOri) mice were obtained from Samtako (Osan-si, Republic of Korea) and randomly divided into eighteen groups (*n* = 8 per group) were used (Table 2). Based on Table 2, mice in each group were immunized intramuscularly with recombinant protein (20 μg/mouse) after mixing with Alhydrogel^®^ adjuvant 2% (1:9 ratio, InvivoGen, San Diego, CA, USA), and control mice (group 1) were injected with phosphate-buffered saline (PBS). All mice received the same booster immunization two weeks later. The mouse sera were collected at two and five weeks post-boost immunization.

An immunogenicity experiment using different adjuvants including Alum (Adju-Phos^®^ adjuvant and Alhydrogel^®^ adjuvant 2%, Invivogen, San Diego, CA, USA), AS04 (MPLA-SM VacciGrade™, Invivogen, San Diego, CA, USA), AS03 (AddaS03™, Invivogen, San Diego, CA, USA), and MF59 (AddaVax™, Invivogen, San Diego, CA, USA) was also conducted in BALB/c mice. Mice were randomly divided to 11 groups (*n* = 6) and were vaccinated intramuscularly with the most immunogenic NiV-vaccine candidate from above and in combination with different adjuvants, and control mice (NC) were injected with PBS (Table 3). As above, a booster immunization was given and sera were collected 2 weeks post-boost immunization. In all, animal experiments, clinical signs such as ruffled fur, pain, distress, and swelling at the injection site, and mortality were monitored daily post-vaccination. All animals were housed and cared for in strict adherence to the guidelines for the care and use of laboratory animals. The animal studies were reviewed and approved by the Animal Experimental Committee of the Laboratory Animal Center, Daegu-Gyeongbuk Medical Innovation Foundation (IACUC number: KMEDI-23022101-01), and the combination of adjuvant mouse studies were approved by the IACUC of the Korea Centers for Disease Control and Prevention (KDCA-IACUC-23-002).

### 2.6. Enzyme-Linked Immunosorbent Assay

Serum samples (1:100 dilution) were tested for specific antibodies against F and/or G glycoproteins of NiV by enzyme-linked immunosorbent assay (ELISA). In brief, the microplates were coated with recombinant proteins, 100 μL/well of 5 μg/mL of respective glycoprotein diluted in coating buffer (Invitrogen, Carlsbad, CA, USA) that were used in immunization and incubated overnight at 4 °C. Plates were washed 6 times with PBS-T and blocked for 1 h at room temperature with 100 μL/well of blocking buffer. Plates were incubated for 1 h, washed, then incubated for 1 h with HRP-conjugated goat anti-mouse IgG (H + L, ThermoFisher Scientific, San Diego, CA, USA) diluted in blocking buffer at room temperature. Plates were developed with 50 μL/well SureBlueTM TMB 1-Peroxidase Substrate (Seracare Life Sciences, Gaithersburg, MD, USA). The assay was performed in triplicate, and the optical density of each well was determined using a microplate reader (Bio-Rad, Hercules, CA, USA) at 450 nm.

### 2.7. Generation of Pseudovirus Expressing Nipah Virus F or G Glycoproteins

The NiV-F or -G pseudoviruses were generated as described elsewhere, with modification [31]. Briefly, NiV Malaysia strain-F- and -G-expressing plasmids, and the VSV packaging system incorporating luciferase fluorescent, were co-transfected to BHK-21 cells using Lipofectamine 2000 (Invitrogen, Carlsbad, CA, USA). VSV-luciferase-NiV pseudovirus containing supernatant of the transfected cells was harvested after 48 h, centrifuged at 1400× *g* for 10 min, filtered with 0.45-µm filter (Millipore, Darmstadt, Germany), and stored in aliquots in −70 °C until used.

### 2.8. Pseudovirus-Based Neutralization Assay

A neutralization assay was performed as described [31]. In brief, VSV-luciferase-NiV pseudovirus was serially diluted with sample sera then incubated for 1 h at 37 °C in duplicates, after which the mixture was added to Vero cells in opaque black 96-well tissue culture plates. A total of 48 h post-incubation, media were removed and 50 µL of luciferase substrate (ONE-Glo™ Luciferase Assay system, Catalog ID: E6120, Promega, Madison, WI, USA) was added to each well. Five min after incubation, the fluorescence was measured at 570 nm using SpectraMax i3x (Molecular devices, San Jose, CA, USA) and the IC50 values were calculated in GraphPad Prism 10.0 (GraphPad Software Inc., San Diego, CA, USA) using non-linear regression (log vs. response).

### 2.9. Statistical Analysis

All data were analyzed and graphs generated using GraphPad Prism 10.0 (GraphPad Software Inc., San Diego, CA, USA) and values expressed as the mean values ± standard deviation (SD). The two-tailed unpaired Student’s t-test or one-way ANOVA test was performed between experimental groups, and a *p*-value of less than 0.05 indicated statistically significant difference.

## 3. Results

### 3.1. Nipah Virus Strain Selection

In order to choose sequences of NiV that could elicit broad immunogenicity against various sub-groups, we analyzed most of the possible full-length sequences in NCBI. A total of 78 and 71 full-length NiV F and G glycoprotein gene sequences were retrieved for phylogenetic analysis, respectively (Supplementary Table S1). Preliminary phylogenetic analysis of the sequences showed that both F and G genes are generally divided into two clusters, NiV-MY and NiV-BD. Additionally, NiV-BD have three different sub-groups, BD1, BD2, and India. Based on this, a consensus sequence for each gene group was acquired, and subsequent sequence similarity and phylogenetic analysis was performed. The acquired NiV-GMY, NiV-GBD, and NiV-F amino acid sequences in the study had homology with the NiV strains of 95.19–100%, 95.69–100%, and 98.54–100%, respectively (Supplementary Table S2). Accordingly, the nucleotide similarity of the consensus sequences for each glycoprotein gene sequence was high (Supplementary Table S3). The amino acid of the derived consensus sequence of the F gene had high similarity to all groups, and thus was chosen to design the vaccine harboring NiV-F. Conversely, amino acid similarity of the consensus sequence of NiV-G gene was more similar to NiV-BD groups and was selected to design NiV-GBD only, while the isolate MK673562, with the highest similarity to all NiV-GMY gene sequences, was chosen to design NiV-GMY. The phylogenetic relationship of the selected antigens for vaccine development to other known NiV strains are shown in Supplementary Figure S1.

### 3.2. Design of NiV-F/G Vaccine Candidates

**Figure 1:**
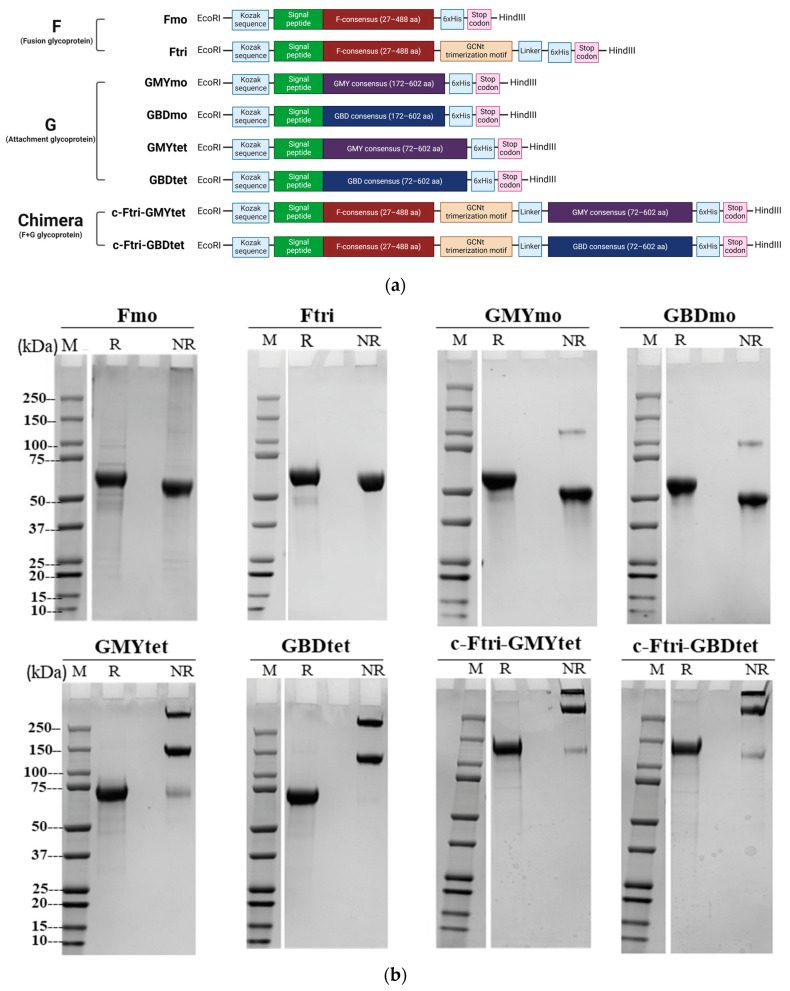

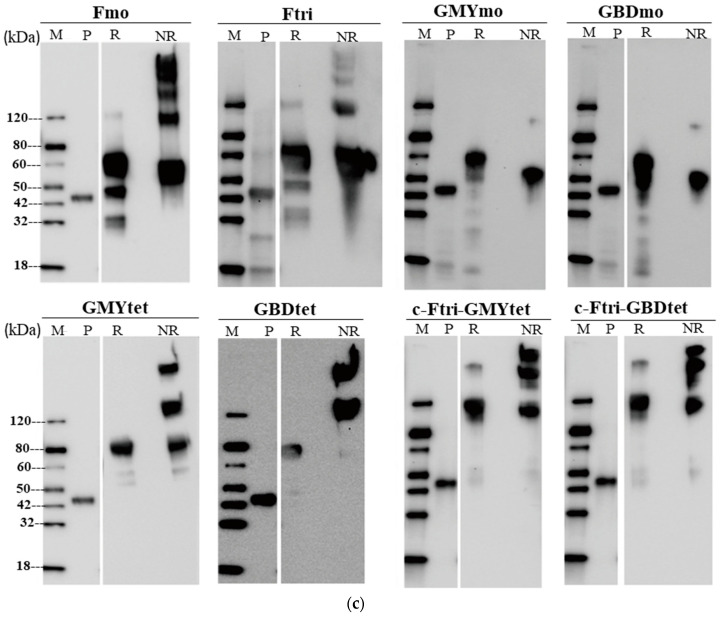
Production and confirmation of recombinant NiV proteins. The recombinant NiV F and G glycoproteins were expressed from the mammalian expression system. (**a**) Schematic representation of the plasmid constructs for NiV recombinant protein expression. (**b**) SDS-PAGE of recombinant NiV F and G glycoproteins. Image was created in Biorender.com with publication and licensing rights. (**c**) Western blot of recombinant NiV F and G glycoproteins. M, molecular size marker; P, multiple-tag positive control (GenScript, Cat. No. M0101, China); R, reducing condition; NR, non-reducing condition; Fm, F glycoprotein monomer; Ftri, F glycoprotein trimer; GMYm, G glycoprotein derived from Malaysia strain monomer; GBDm, G glycoprotein derived from Bangladesh strain monomer; GMYtet, G glycoprotein derived from Malaysia strain tetramer; GBDtet, G glycoprotein derived from Bangladesh strain tetramer; c, chimeric.

Based on the sequences from the phylogenetic analysis, three sequences (NiV-F concensus, NiV-GBD consensus, and NiV-GMY) were selected to design plasmid constructs to express NiV-pre-F and NiV-G glycoproteins in monomeric, multimeric, and chimeric forms. A codon of each nucleotide was optimized and cloned to the mammalian expression vector to generate the proteins (Table 1). Multimeric structures of the NiV-pre-F and NiV-G immunogens were constructed with a trimerization motif, and chimeras expressing both NiV-pre-F and G were constructed by directly linking the C-terminus region of NiV-pre-F to the trimerization motif which was linked to the N-terminus of NiV-G residues via a linker (GGGGS) (Figure 1a). All sequences derived from this study were submitted to GenBank with accession numbers OR947674-947676 (Table 1).

### 3.3. Expression of Recombinant NiV Proteins

Eight recombinant NiV-G and -pre-F glycoproteins were constructed (Figure 1a), and the glycoproteins were expressed in HD-293F suspension cells and purified by affinity chromatography. The expression and size of the recombinant proteins were confirmed by SDS-PAGE (Figure 1b), as well as in the reducing and non-reducing conditions by western blot (Figure 1c). In order to further validate the antibody reactivity of the expressed recombinant proteins, western blot was performed using commercially available NiV antibodies (Catalog ID: MAB12307, DMAB001, ab63557). The findings affirm the reactivity of the produced recombinant proteins NiV-pre-F and NiV-G (monomer, multimer, or chimeric) from this study to their respective monoclonal antibodies (NiV-F: MAB12307, NiV-G: DMAB001) and polyclonal NiV antibody (ab63557).

### 3.4. Immunogenity of Recombinant NiV Proteins

**Figure 2:**
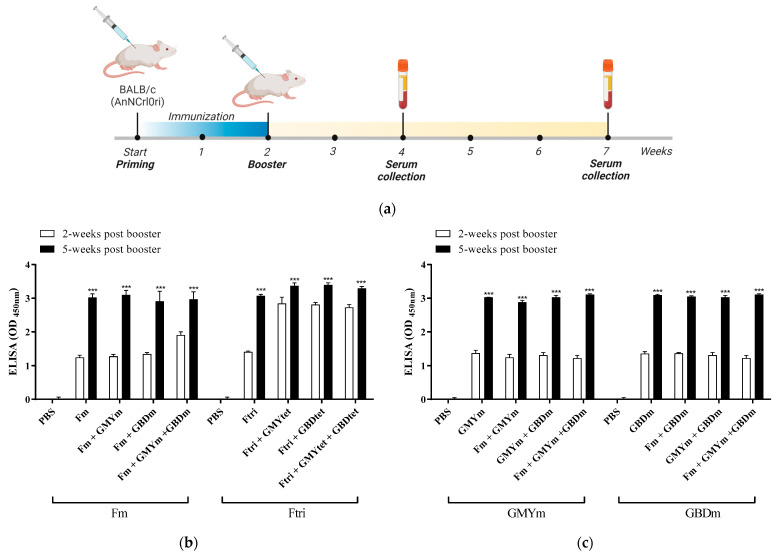

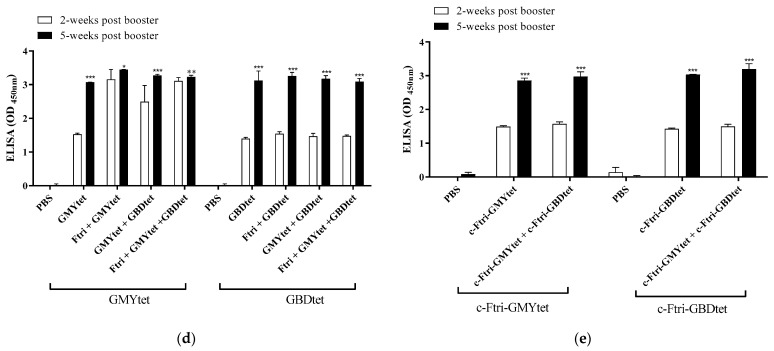
Immunogenicity of the NiV-F and NiV-G vaccine candidates in BALB/c mice. (**a**) Schematic timeline of the immunization and serum collection schedule in mice. Mice were vaccinated intramuscularly twice at two-week intervals, and the serum was collected two and five weeks post-booster vaccination to determine humoral response by ELISA. Image was created in Biorender.com with publication and licensing rights. Specific IgG antibodies for (**b**) NiV-F, (**c**,**d**) NiV-G, and (**e**) NiV-F/G. The specific antibodies in the serum (*n* = 8) were measured by ELISA, and the values are presented as mean absorbance at 450 nm (OD 450 nm) ± SD. Statistical significance between groups for each time point collection (2 and 5 weeks post-booster) was performed using unpaired *t*-test. * *p* < 0.05, ** *p* < 0.01, *** *p* < 0.001.

To evaluate the immunogenicity of the eight recombinant proteins, specific pathogen-free (SPF) male BALB/c (AnNCrlOri) mice aged six-weeks old were immunized twice following their designated group (Figure 2a, Table 2). For the duration of the experiment, no mortality was recorded in any group, and immunized mice did not show any clinical signs. Serum samples were collected two and five weeks post-booster vaccination in all groups, and the NiV-specific antibody titers were evaluated using ELISA (Figure 2b–e). Notably, in all groups, specific antibodies were detected as early as two weeks post-booster vaccination, and the specific IgG antibodies in the mouse serum increased significantly at five weeks post-vaccination, compared to after two weeks for all groups (Figure 2b–e) These findings suggest that all NiV-vaccine candidates were able to elicit effective immune response in mice.

### 3.5. Neutralization Efficacy of Recombinant NiV Proteins

**Figure 3:**
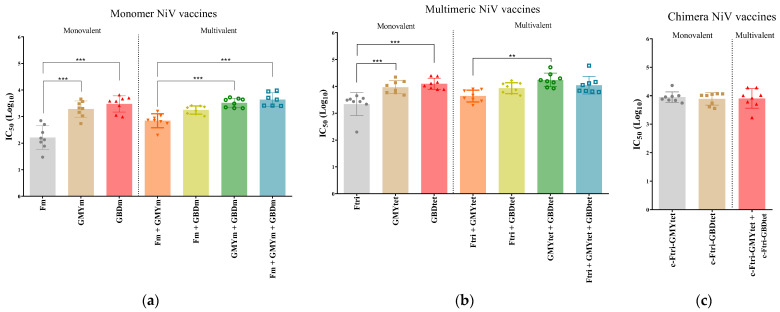
Neutralization of VSV-luciferase-NiV-pseudovirus. Neutralization assays were performed on individual mouse sera collected at five weeks post-booster vaccination from mice vaccinated with recombinant NiV vaccine harboring NiV-pre-F and NiV-G glycoprotein in (**a**) monomeric structure, (**b**) multimeric structure, and (**c**) chimera, either administered as monovalent or multivalent. The IC50 for each sample were calculated by curve fitting and non-linear regression in GraphPad Prism, and indicated in Log10 values. Statistical significance between monovalent and multivalent groups for each vaccine structure (monomer, multimer, and chimera) was performed using one-way ANOVA and Tukey’s multiple comparison test. Each shape within group represent individual values of mouse sera, bars indicate mean values of the group, and error bars are ± standard deviation. ** *p* < 0.001, *** *p* < 0.001.

To evaluate the neutralizing antibodies elicited by the different NiV-vaccine candidates, a neutralization assay using VSV-luciferase-NiV pseudovirus was employed. In all vaccinated groups, similar to the ELISA result, neutralization antibody was present (Figure 3). Amongst monomer vaccines administered alone, both GMYm (mean IC50Log10: 3.28) and GBDm (mean IC50Log10: 3.47) vaccines elicited more significant neutralizing antibody than Fm (mean IC50Log10: 2.21). Conversely, in monomer-structured vaccine candidates administered as multivalent, bivalent GMYm+ GBDm (mean IC50Log10: 3.52) and trivalent Fm + GMYm + GBDm (mean IC50Log10: 3.64) produced the highest neutralizing antibodies (Figure 3a). In summary, monomer-structure NiV recombinant vaccine candidates had a mean IC50log10 value between 2.21–3.64. On the other hand, the mean IC50Log10 on multimeric structure vaccines administered alone was significantly higher in sera collected from GMYtet (mean IC50Log10: 3.97)-vaccinated and GBDtet (mean IC50Log10: 4.10)-vaccinated, than in Ftri (mean IC50Log10: 3.38)-vaccinated, mice. Furthermore, in multimeric vaccine candidates given in combination, GMYtet + GBDtet (mean IC50Log10: 4.24) elicited the highest neutralizing antibodies, followed by Ftri + GBDtet (mean IC50Log10: 3.93) and Ftri +GMY tet + GBDtet (mean IC50Log10: 4.05) (Figure 3b). Compared to monomer vaccine candidates, the multimeric NiV vaccine candidates have a mean IC50log10 value of 3.38–4.24. Lastly, neutralizing antibodies were also high in mice vaccinated with chimeric vaccine candidates, but no significant difference was found between groups (mean IC50Log10: 3.89–3.95) (Figure 3c). These results indicated that all the vaccine candidates induced a potent immune response in mice against NiV, however, a stronger response was observed in multimeric-structure vaccines administered as multivalent (bivalent or trivalent) than when given in their monomeric structures and alone. Moreover, when comparing all groups, bivalent GMYtet + GBDtet elicited the most potent neutralizing activity. Multiple comparison of significance in all groups is summarized in Supplementary Table S4.

### 3.6. Immunogenicity of Bivalent and Trivalent NiV Vaccines Using Different Adjuvants

**Figure 4:**
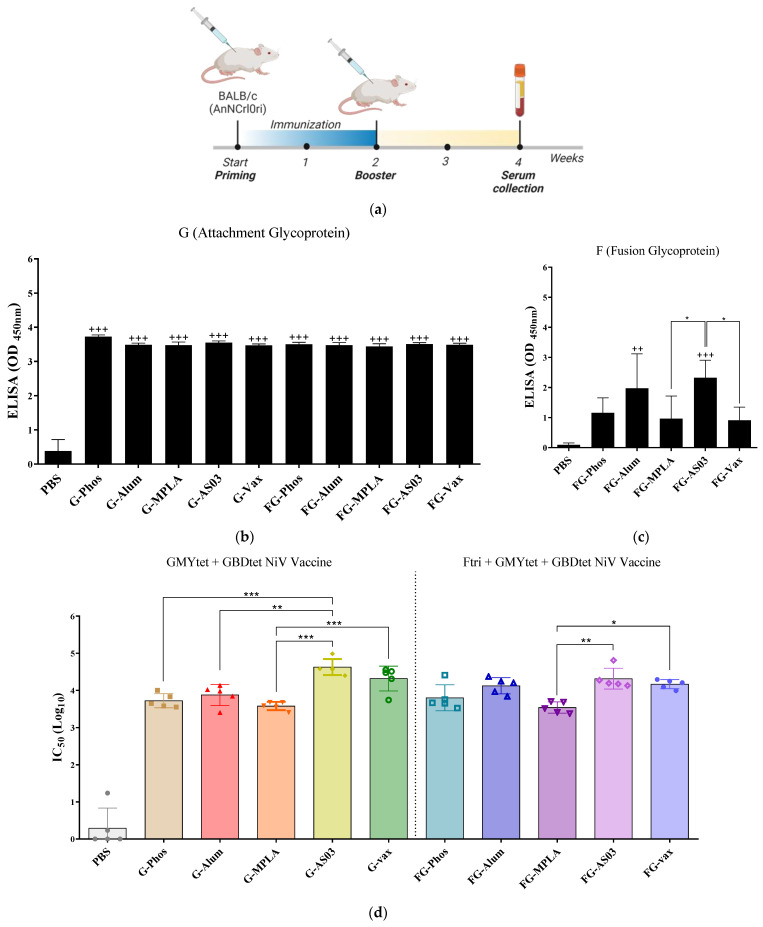
Immunogenicity of vaccines with different adjuvants. (**a**) Schematic timeline of the immunization and serum collection schedule in mice. Mice were vaccinated intramuscularly twice with bivalent (GMYtet + GBDtet) and trivalent (Ftri + GMYtet + GBDtet) in different adjuvants at two-week intervals, and the serum was collected two weeks post-booster vaccination to determine humoral response by ELISA. Image was created in Biorender.com with publication and licensing rights. Specific IgG antibodies for (**b**) NiV-G and (**c**) NiV-F. The specific antibodies in the serum (*n* = 6) were measured by ELISA at two weeks post-booster immunization, and the values are presented as mean absorbance at 450 nm (OD 450nm) ± SD. Statistical significance between groups was performed using one-way ANOVA, and Tukey’s multiple comparison test was used to compare significance compared to the control (++ *p* < 0.01; +++, *p* < 0.001) and between groups (* *p* < 0.05). (**d**) Neutralization assays were performed on sera collected at two weeks post-booster vaccination from vaccinated mice. Each shape within group represent individual values of mouse sera, and error bars are ± standard deviation. * *p* < 0.05, ** *p* < 0.01, *** *p* < 0.001.

To determine whether adjuvants affected the immunogenicity of the most immunogenic multivalent vaccine candidates from the previous experiment, mice were vaccinated twice with bivalent (GMYtet + GBDtet) and trivalent (Ftri + GMYtet + GBDtet) vaccines in different adjuvants (Phos, Alum, MPLA, AS03 and Vax) intramuscularly at two-week intervals (Table 3), and sera were collected for ELISA and pseudovirus neutralization assay (Figure 4). All vaccinated mice survived and showed no clinical signs. The specific IgG for NiV-G was detected at significantly high levels in the sera of all vaccinated groups, regardless of adjuvant, compared to the PBS group (Figure 4b). Conversely, antibodies for NiV-F were detected in all groups but were only significantly found in NiV-vaccinated mice adjuvanted with FG-Alum and FG-AS03 (Figure 4c). Interestingly, FG-AS03 elicited more response than FG-MPLA and FG-Vax. Neutralizing antibody was detected in all groups. In mice immunized with bivalent (GMYtet + GBDtet) vaccine, neutralizing antibodies were highest when the vaccine was adjuvanted with G-AS03 (mean IC50Log10: 4.67) followed by G-vax, G-Alum, G-Phos, and G-MPLA in decreasing order with a mean IC50Log10 of 4.32, 3.88, 3.72, and 3.58, respectively. On the other hand, in trivalent vaccine (Ftri + GMYtet + GBDtet)-immunized groups, the mean IC50Log10 were 4.41, 4.17, 4.12, 3.8, and 3.54 for FG-AS03, FG-vax, FG-Alum, FG-Phos, and FG-MLPA, respectively. The results from the pseudovirus neutralization assay consistently showed that neutralizing antibodies were highest when administered with AS03, and least when administered with MPLA (Figure 4d). Collectively, the results from this experiment indicated that adjuvants could optimize the immune response elicited by the recombinant structure-based mammalian-expressed bivalent (GMYtet + GBDtet) and trivalent NiV vaccines (Ftri + GMYtet + GBDtet), particularly when administered with AS03, followed by Vax, Alum, Phos, and MPLA.

## 4. Discussion

NiV is a high-priority zoonotic pathogen first reported in Malaysia that is now causing recurrent outbreaks in Bangladesh and India, with high fatality rates and debilitating outcomes. There is a growing list of NiV-vaccine candidates utilizing different methods with pre-clinical trial results of varying levels of protection against NiV infection [28,38,39]. However, there is not yet an approved vaccine against NiV in humans, which necessitates a continued effort to develop novel, effective vaccines that can induce a potent and long-lived immune response against NiV infection that are multivalent, reproducible, stable, and safe. Protein structure is a powerful tool when applied to design and development of vaccine immunogens [40,41]. Here, the vaccine candidates were designed based on knowledge from previous immunogen design efforts for paramyxoviruses and RSV, to stabilize NiV F glycoprotein in its pre-F conformation [33,34]. The lead vaccine design is increased if antigen design strategies can be applied to other strains within a virus family or genus, as previously demonstrated for henipaviruses and coronaviruses [31,35]. This study designed mammalian-expressed NiV vaccine candidates in monomeric, multimeric, and chimeric forms, and evaluated their immunogenicity in mice. Subsequently, the effects of different adjuvants were determined on the potential vaccine candidates.

NiVs are generally classified into the Malaysia (NiV-MY) strain and the Bangladesh (NiV-BD) strain [3]. Although they share high homology and are essentially indistinguishable, they form distinct phylogenetic groups and can cause different pathogenicity outcomes in their hosts and viral transmission [42,43,44]. The development of vaccine immunogens requires critical selection of the antigen. NiV-G and NiV-F glycoproteins are two membrane glycoproteins that are known targets of vaccine-elicited neutralizing antibodies and, thus, were the focus of this study [45]. Based on the phylogenetic analysis of all available full-length sequences of NiV-F and NiV-G, three sequences based on the consensus sequences were chosen to design recombinant proteins as vaccine candidates. The sequences were optimized to express a different stable structure of the proteins, including the addition of the GCN4 trimerization motif, mutations, and linker based on previous reports [31].

Recombinant proteins were successfully expressed in mammalian cells and were found to elicit specific antibody production as early as two weeks post-booster vaccination, with higher production at five weeks after booster vaccination. Moreover, antibodies in the sera of mice were able to neutralize VSV-luciferase-NiV pseudovirus at varying levels. Notably, monomer NiV-F elicited a lower response than its NiV-Gm counterpart, or when NiV-Fm was administered in combination with NiV-G or alone in NiV-Ftri form. Conversely, NiV-G are immunogenic, whether administered as a monovalent in monomer or multimer structure, or as multivalent, with the highest neutralizing antibodies in all groups induced when NiV-GMY and NiV-GBD were in their multimeric form and administered together as bivalent (GMYtet + GBDtet) or trivalent with NiV-Ftri (Ftri + GMYtet + GBDtet). These findings are consistent with the protective efficacy of mammalian-expressed recombinant soluble NiV-G in CpG/Alum adjuvant [46]. Additionally, the recombinant chimeras of NiV-F/G in this study were also able to induce potent neutralizing antibodies, but to a lesser extent compared to GMYtet + GBDtet and Ftri + GMYtet + GBDtet. The superiority of bivalent or trivalent NiV-G multimeric over chimeras contrast with a previous study [31]. While both NiV F and G glycoproteins are key antigens known to elicit a protective immune response against NiV infection, G is considered to be the most dominant neutralizing target [31,47]. In a previous report, pigs were found to have better protection against NiV infection when vaccinated with a combination of a canarypox virus-based recombinant NiV F and G proteins, compared to an immunization with individual F or G protein [48].

In sub-unit vaccines, adjuvants play a significant role in eliciting protective immune response. Hence, the most immunogenic bivalent vaccine (GMYtet + GBDtet) and trivalent vaccine (Ftri + GMYtet + GBDtet) from the first experiment were selected for further immunogenicity trials in mice, using different adjuvants to evaluate whether these adjuvants could optimize vaccine-mediated immune response. A list of common adjuvants used in licensed vaccines includes aluminum, virosomes, AS04, MF59, AS03, thermo-reversible oil-in-water, and ISA51 [49]. In this study, four different adjuvant types were compared. The action of AS03 and AS04 generally relates to the induction of innate immune cells and effectors, while aluminium and MF59 can elicit a robust Th2- and Th1-driven immune response, respectively [46,49,50]. Results from the adjuvant experiment in this study demonstrated that, while all adjuvants can elicit a robust immune response, recombinant vaccines administered with AS03 induced significantly more potent vaccine-induced immune response, suggesting its application as preferential adjuvant for NiV recombinant vaccine candidates. In a previous study, AS03-adjuvanted DNA vaccines were found to induce robust humoral and cellular immune response against SARS-CoV-2 [51]. Moreover, the use of AS03-adjuvanted coronavirus virus-like particle (CoVLP) was found to be safe and it was able to induce a highly tolerated immune response for at least 6 months against SARS-CoV-2 [52].

## 5. Conclusions

While all vaccine candidates in this study were found to be immunogenic, GMYtet + GBDtet-vaccinated mice showed significantly stronger neutralizing antibodies, highlighting its ideal potential as the lead mammalian-expressed recombinant vaccine candidate to be further explored and optimized. Moreover, AS03-adjuvanted mammalian-expressed recombinant NiV vaccines are generally more immunogenic than when administered with Alum, AS04, or MF59. However, the exact protective effects, including the underlying mechanism and related host–immune response of these vaccine candidates, is yet to be elucidated in in vivo infection models using live virus challenges. Collectively, findings from this study demonstrated the potential of the mammalian-expressed NiV recombinant proteins as NiV vaccine candidates, and highlighted the critical role of adjuvants in optimizing the efficacy of mammalian-expressed recombinant NiV vaccines.

## Figures and Tables

**Table 1:** Plasmid design to express recombinant proteins used in this study.

Name	Target Sequence	Structure	Mutation	Tag	Multimeric Domain	Accession Number
NiV_Fmo	F	Monomer	I114C, L104C, S191P, L172F	6× his	-	In this study (OR947674)
NiV_Ftri	F	Trimer	I114C, L104C, S191P, L172F	6× his	GCN4	
NiV_GMYm	G	Monomer	-	6× his	-	In this study (OR947675)
NiV_GBDm	G	Monomer	-	6× his	-	In this study (OR947676)
NiV_GMYtet	G	Tetramer	-	6× his	-	In this study (OR947675)
NiV_GBDtet	G	Tetramer	-	6× his	-	In this study (OR947676)
C_Ftri_GMYtet	F + G	Chimeric ^†^	-	6× his	GCN4	
C_Ftri_GBDtet	F + G	Chimeric ^†^	-	6× his	GCN4	

F, fusion glycoprotein; G, attachment glycoprotein; GMY, attachment glycoprotein of Malaysia strain; GBD, attachment glycoprotein of consensus sequence Bangladesh/India strain; m, monomer; tri, trimer; tet, tetramer; Chimeric ^†^ structure harboring trimer and tetramer for F and G proteins, respectively.

**Table 2:** Experimental design of the eight NiV vaccines for immunogenicity study in BALB/c mice.

Group	Vaccine	Dose	Description	Target	Route	Sample Size (*n*)
1	PBS	20 μg	Control		IM	8
2	Fm	20 μg	F monomer	F	IM	8
3	GMYm	20 μg	GMY monomer	GMY	IM	8
4	GBDm	20 μg	GBD monomer	GBD	IM	8
5	Fm + GMYm	20 μg + 20 μg	F monomer + GMY monomer	F, GMY	IM	8
6	Fm + GBDm	20 μg + 20 μg	F monomer + GMY monomer	F, GBD	IM	8
7	Ftri	20 μg	F trimer	F	IM	8
8	GMYtet	20 μg	GMY tetramer	GMY	IM	8
9	GBDtet	20 μg	GBD tetramer	GBD	IM	8
10	Ftri + GMYtet	20 μg + 20 μg	F trimer + GMY tetramer	F, GMY	IM	8
11	Ftri + GBDtet	20 μg + 20 μg	F trimer + GBD tetramer	F, GBD	IM	8
12	c-Ftri-GMYtet	20 μg	Chimeric F trimer-GMY tetramer	F, GMY	IM	8
13	c-Ftri-GBDtet	20 μg	Chimeric F trimer- GBD tetramer	F, GBD	IM	8
14	GMYm + GBDm	20 μg + 20 μg	GMY monomer + GBD monomer	GMY, GBD	IM	8
15	Fm + GMYm + GBDm	20 μg + 20 μg + 20 μg	F monomer + GMY monomer + GBD monomer	F, GMY, GBD	IM	8
16	GMYtet + GBDtet	20 μg + 20 μg	GMY tetramer + GBD tetramer	GMY, GBD	IM	8
17	Ftri + GMYtet + GBDtet	20 μg + 20 μg + 20 μg	F trimer + GMY tetramer + GBD tetramer	F, GMY, GBD	IM	8
18	c-Ftri-GMYtet + c-Ftri-GBDtet	20 μg + 20 μg	Chimeric F trimer-GMY tetramer + Chimeric F trimer- GBD tetramer	F, GMY, GBD	IM	8

PBS, phosphate buffer saline; F, fusion glycoprotein; GMY, attachment glycoprotein of Malaysia strain; GBD, attachment glycoprotein of Bangladesh/India strain; c, chimeric; IM, intramuscular.

**Table 3:** Experimental design for immunogenicity study using different adjuvants.

Group	Vaccine	NiV-Vaccine	Dose	Adjuvant	Route	Sample Size (*n*)
1	PBS				IM	6
2	G-Phos	GMYtet + GBDtet	20 µg + 20 µg	Phos	IM	6
3	G-Alum	GMYtet + GBDtet	20 µg + 20 µg	Alum	IM	6
4	G-MPLA	GMYtet + GBDtet	20 µg + 20 µg	MPLA	IM	6
5	G-AS03	GMYtet + GBDtet	20 µg + 20 µg	AS03	IM	6
6	G-Vax	GMYtet + GBDtet	20 µg + 20 µg	Vax	IM	6
7	FG-Phos	Ftri + GMYtet + GBDtet	20 µg + 20 µg + 20 µg	Phos	IM	6
8	FG-Alum	Ftri + GMYtet + GBDtet	20 µg + 20 µg + 20 µg	Alum	IM	6
9	FG-MPLA	Ftri + GMYtet + GBDtet	20 µg + 20 µg + 20 µg	MPLA	IM	6
10	FG-AS03	Ftri + GMYtet + GBDtet	20 µg + 20 µg + 20 µg	AS03	IM	6
11	FG-Vax	Ftri + GMYtet + GBDtet	20 µg + 20 µg + 20 µg	Vax	IM	6

Alum, Alhydrogel^®^ adjuvant 2%; Ftri, fusion protein trimer structure; GMYtet, glycoprotein of Malaysian strain tetramer structure; GBDtet, glycoprotein of Bangladesh/India strain tetramer structure; IM, intramuscular; MPLA, MPLA-SM VacciGrade™; Phos, Adju-Phos^®^ adjuvant; AS03, AddaS03™; Vax, AddaVax™.

## Data Availability

Data used in all analyses are included in this article and its Supplementary Materials. All other inquiries can be directed to the corresponding authors.

## Supplementary Materials

The following supporting information can be downloaded at: https://www.mdpi.com/article/10.3390/vaccines12090999/s1, Figure S1: phylogenetic tree of NiV F and G genes. (a) Phylogenetic analysis of NiV-G genes with NiV-GMY and NiV-GBD. The consensus and representative sequences were pointed red arrows. (b) Phylogenetic analysis of NiV-F genes with NiV-F. The ancestor sequence was pointed red arrows. For each gene, a maximum likelihood tree of 71 full-length sequences of NiV strains was generated in MEGA using the HKY model with 1000 bootstrap. All positions with less than 95% site coverage were eliminated. Table S1: list of NiV reference sequences used for NiV-F and NiV-G gene sequence analyses. Table S2: similarity of NiV-F and NiV-G amino acid sequences. Table S3: similarity of NiV-F and NiV-G nucleotide sequences. Table S4: test of significance between groups using Dunn’s multiple comparison test.
