# Supplementary material for: Immunogenicity and Neutralization of Recombinant Vaccine Candidates Expressing F and G Glycoproteins against Nipah Virus
Source: Vaccines (Basel). 2024 Aug 31;12(9):999. doi: 10.3390/vaccines12090999 (PMC11436239; doi:10.3390/vaccines12090999)
Supplement: Supplementary file 1 [file vaccines-12-00999-s001.zip › Supplementary Table S1. List of isolates.pdf]

Supplementary Table S1. List of NiV isolates used for NiV-F and NiV-G gene sequence analysis.

F gene	G gene
>NC_002728.1*	>NC_002728.1
>KY425655.1	>KY425655.1
>KY425646.1	>KY425646.1
>MK673563.1	>MK673563.1
>MK673561.1	>MK673561.1
>MK673560.1	>MK673560.1
>MK673559.1	>MK673559.1
>MK673558.1	>MK673558.1
>MK673562.1	>MK673562.1
>AJ627196.1	>AJ627196.1
>MK673564.1	>MK673564.1
>MK673565.1	>MK673565.1
>MK673582.1	>MK673582.1
>MK673568.1	>MK673568.1
>MK673591.1	>MK673591.1
>MK673581.1	>MK673581.1
>AY988601.1	>AY988601.1
>FN869553.1	>FN869553.1
>MK673573.1	>MK673573.1
>MK673566.1	>MK673566.1
>JN808864.1	>JN808864.1
>MK673577.1	>MK673577.1
>MK673574.1	>MK673574.1
>MK673572.1	>MK673572.1
>MK673571.1	>MK673571.1

>MK673567.1	>MK673567.1
>MK575070.1	>MK575070.1
>MK575069.1	>MK575069.1
>MK575068.1	>MK575068.1
>MK575067.1	>MK575067.1
>MK575066.1	>MK575066.1
>MK575065.1	>MK575065.1
>MK575064.1	>MK575064.1
>MK575062.1	>MK575062.1
>MK575061.1	>MK575061.1
>MK575060.1	>MK575060.1
>MK673590.1	>MK673590.1
>MK673589.1	>MK673589.1
>MK673579.1	>MK673579.1
>JN808857.1	>JN808857.1
>FJ513078.1	>FJ513078.1
>MK673583.1	>MK673583.1
>MK673570.1	>MK673570.1
>MW535746.1	>MW535746.1
>MK673575.1	>MK673575.1
>MN549411.1	>MN549411.1
>MH523642.1	>MH523642.1
>MH523640.1	>MH523640.1
>MK673584.1	>MK673584.1
>MK673576.1	>MK673576.1
>MN549409.1	>MN549409.1
>MK673592.1	>MK673592.1
>MK673585.1	>MK673585.1
>MK673588.1	>MK673588.1

>MH396625.1	>MH396625.1
>MK336156.1	>MK336156.1
>MK336155.1	>MK336155.1
>MH523641.1	>MH523641.1
>MK575063.1	>MK575063.1
>MN549403.1	>MN549403.1
>AJ564623.1	>AJ564623.1
>AJ564621.1	>AJ564621.1
>AJ564622.1	>AJ564622.1
>AY029768.1	>AY029768.1
>AF212302.2	>AF212302.2
>JN808863.1	>JN808863.1
>AF376747.1	>AF376747.1
>AY029767.1	>AY029767.1
>MK673578.1	>MK673578.1
>MK801755.1	>MK801755.1
>MK673580.1	>HM545086.1
>MK673587.1	>MK673591.1
>MK673586.1	
>MN549402.1	
>MN549410.1	
>MN549406.1	
>MN549405.1	
>MN549408.1	

*: Reference sequence
