# Supplementary material for: Immunogenicity and Neutralization of Recombinant Vaccine Candidates Expressing F and G Glycoproteins against Nipah Virus
Source: Vaccines (Basel). 2024 Aug 31;12(9):999. doi: 10.3390/vaccines12090999 (PMC11436239; doi:10.3390/vaccines12090999)
Supplement: Supplementary file 1 [file vaccines-12-00999-s001.zip › Supplementary Table S2. Amino acid similarity.pdf]

Supplementary Table S2. Similarity of the NiV-F and NiV-G amino acid sequences.

Gene		NiV-MY(%)	NiV-BD1(%)	NiV-BD2(%)	NiV-India(%)	Total(%)
F	NiV F (OR947674)	98.54-98.90	99.82-100	99.27-100	99.45-99.82	98.54-100
G	NiV-MY (MK673562.1)	98.51-100	95.36-95.69	95.36-95.85	95.19-95.52	95.19-100
	NiV-BD (OR947675)	95.69-96.02	99.67-100	99.67-100	98.67-99.00	95.69-100

F, Fusion glycoprotein; G, Attachment glycoprotein; MY, Malaysia strain; BD, Bangladesh/India strain
