# Supplementary material for: Immunogenicity and Neutralization of Recombinant Vaccine Candidates Expressing F and G Glycoproteins against Nipah Virus
Source: Vaccines (Basel). 2024 Aug 31;12(9):999. doi: 10.3390/vaccines12090999 (PMC11436239; doi:10.3390/vaccines12090999)
Supplement: Supplementary file 1 [file vaccines-12-00999-s001.zip › Supplementary Table S3. Nucleotide sequences similarity.pdf]

Supplementary Table S3. Similarity of the NiV-F and NiV-G nucleotide sequences.

Gene		NiV-MY(%)	NiV-BD1(%)	NiV-BD2(%)	NiV-India(%)	Total(%)
F	NiV F (OR947674)	93.97-94.33	98.90-99.27	98.90-99.33	98.84-99.09	93.97-99.33
G	NiV-MY (MK673562.1)	98.23-99.94	92.87-93.03	92.59-92.98	92.48-92.70	92.48-99.94
	NiV-BD (OR947675)	92.81-93.20	99.50-99.78	99.39-99.83	98.12-98.40	92.81-99.83

F, Fusion glycoprotein; G, Attachment glycoprotein; MY, Malaysia strain; BD, Bangladesh/India strain
