# Supplementary material for: Immunogenicity and Neutralization of Recombinant Vaccine Candidates Expressing F and G Glycoproteins against Nipah Virus
Source: Vaccines (Basel). 2024 Aug 31;12(9):999. doi: 10.3390/vaccines12090999 (PMC11436239; doi:10.3390/vaccines12090999)
Supplement: Supplementary file 1 [file vaccines-12-00999-s001.zip › Supplementary Table S4. Multiple Comparison test.pdf]

Supplementary Table S4. Test of significannce between groups using Dunn's Multiple comparison test.

	**Fm**	**GMYm**	**GBDm**	**Fm + GMYm**	**Fm + GBDm**	**GMYm + GBDm**	**Fm + GMYm + GBDm**	**Ftri**	**GMYtet**	**GBDtet**	**Ftri + GMYtet**	**Ftri + GBDtet**	**GMYtet + GBDtet**	**Ftri + GMYtet + GBDtet**	**c-Ftri-GMYtet**	**c-Ftri-GBDtet**	**c-Ftri-GMYtet + c-Ftri-GBDtet**
PBS	ns	ns	ns	ns	ns	ns	ns	ns	***	***	ns	***	***	***	***	***	***
Fm		ns	ns	ns	ns	ns	ns	ns	**	***	ns	***	***	***	***	***	***
GMYm			ns	ns	ns	ns	ns	ns	ns	*	ns	ns	**	ns	ns	ns	ns
GBDm				ns	ns	ns	ns	ns	ns	ns	ns	ns	ns	ns	ns	ns	ns
Fm + GMYm					ns	ns	ns	ns	**	***	ns	*	***	**	*	*	*
Fm + GBDm						ns	ns	ns	ns	*	ns	ns	**	ns	ns	ns	ns
GMYm + GBDm							ns	ns	ns	ns	ns	ns	ns	ns	ns	ns	ns
Fm + GMYm + GBDm								ns	ns	ns	ns	ns	ns	ns	ns	ns	ns
Ftri									ns	ns	ns	ns	*	ns	ns	ns	ns
GMYtet										ns	ns	ns	ns	ns	ns	ns	ns
GBDtet											ns	ns	ns	ns	ns	ns	ns
Ftri + GMYtet												ns	ns	ns	ns	ns	ns
Ftri + GBDtet													ns	ns	ns	ns	ns
GMYtet + GBDtet														ns	ns	ns	ns
Ftri + GMYtet + GBDtet															ns	ns	ns
c-Ftri-GMYtet																ns	ns
c-Ftri-GBDtet																	ns

PBS, Phosphate Buffer Saline; F, Fusion protein; GMY, Glycoprotein of Malaysian strain; GBD, Glycoprotein of Bangladesh/India strain; c, chimera; *$p < 0.05$; **$p < 0.01$; ***$p < 0.001$; ns, not significant
