# Supplementary figures and images for: Immunogenicity and Neutralization of Recombinant Vaccine Candidates Expressing F and G Glycoproteins against Nipah Virus
Source: Vaccines (Basel). 2024 Aug 31;12(9):999. doi: 10.3390/vaccines12090999 (PMC11436239; doi:10.3390/vaccines12090999)

## Slide 1
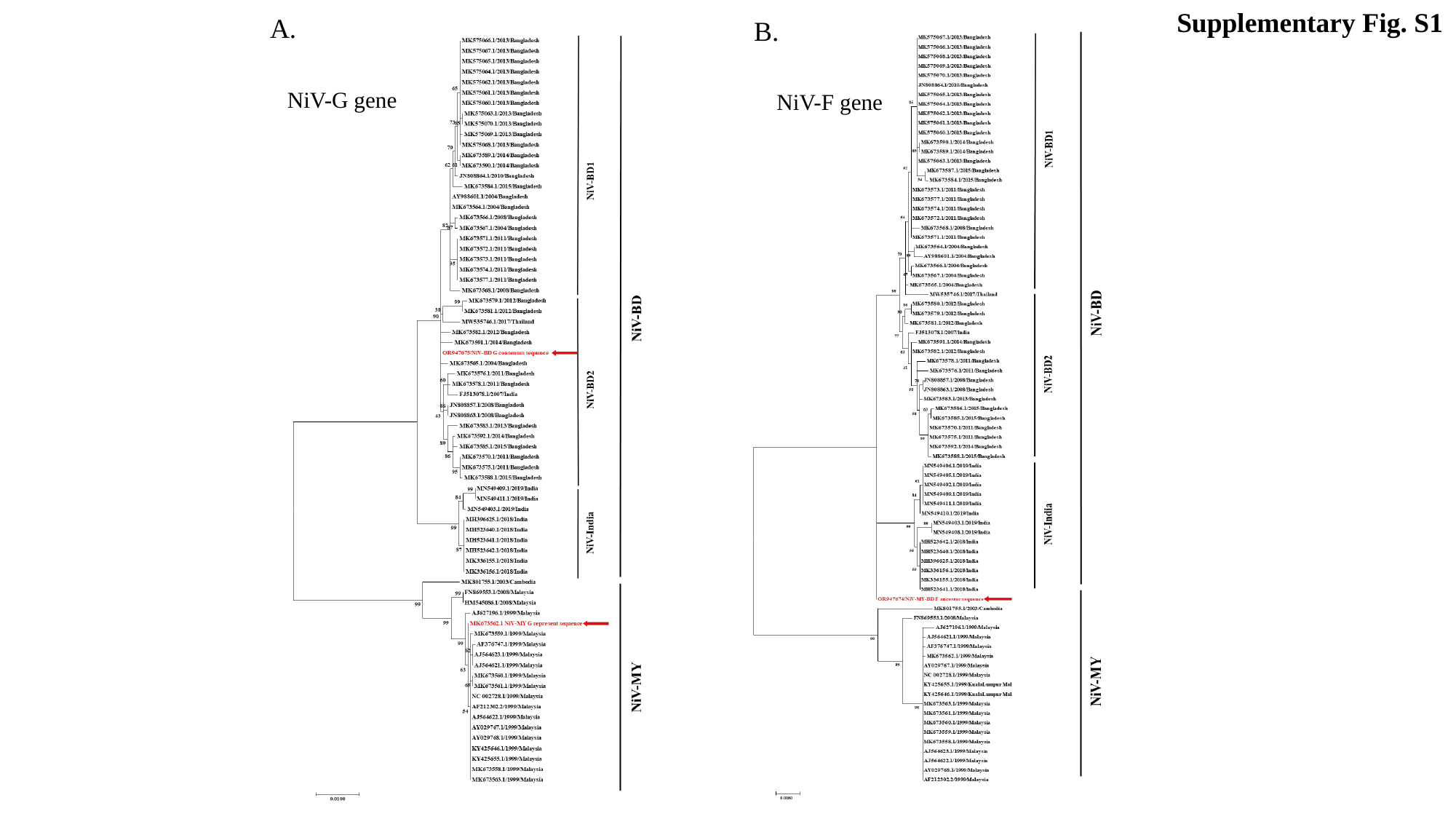

Supplementary Fig. S1
A.
NiV-G gene
B.
NiV-F gene

Supplement: Supplementary file 1 [file vaccines-12-00999-s001.zip › Supplementary Figure S1 Phylogenetic analysis of F and G.pptx]
